# Maintenance Strategies in High-Risk Myeloma: A Multicenter Comparison of Bortezomib–Lenalidomide Versus Lenalidomide Alone: A USMIRC Multicenter Analysis

**DOI:** 10.3390/curroncol33030164

**Published:** 2026-03-13

**Authors:** Sruthi P. Ramanan, Oyepeju F. Abioye, Shebli Atrash, Jianzheng Wu, Dinesh Pal Mudaranthakam, Anita Mazloom, Omar Alkharabsheh, Mansi R. Shah, Zahra Mahmoudjafari, Jordan Snyder, Muhammad Umair Mushtaq, Forat Lutfi, Jeries Kort, Al-Ola Abdallah, Prerna Mewawalla

**Affiliations:** 1 Division of Hematology and Cellular Therapy, AHN Cancer Institute, Allegheny Health Network, Pittsburgh, PA 15212, USA; prerna.mewawalla@ahn.org; 2 Levine Cancer Center, Atrium Health, Wake Forest University School of Medicine, Charlotte, NC 28204, USA; shebli.atrash@advocatehealth.org; 3 Department of Biostatistics & Data Science, University of Kansas Medical Center, Kansas City, KS 66103, USA; jwu3@kumc.edu (J.W.); dmudaranthakam@kumc.edu (D.P.M.); 4 Division of Hematology and Oncology, Mitchell Cancer Institute, University of South Alabama, Mobile, AL 36604, USA; amazloom@health.southalabama.edu (A.M.); alkharor@ucmail.uc.edu (O.A.); 5 Division of Hematology and Oncology, University of Cincinnati, Cincinnati, OH 45267, USA; 6 Division of Blood Disorders, Rutgers Cancer Institute, New Brunswick, NJ 08903, USA; shahmr@rutgers.edu; 7 Division of Hematologic Malignancies & Cellular Therapeutics, University of Kansas Medical Center, Westwood, KS 66205, USA; zmahmoudjafari@kumc.edu (Z.M.); jsnyder1@kumc.edu (J.S.); mmushtaq@kumc.edu (M.U.M.); flutfi@kumc.edu (F.L.); jkort@kumc.edu (J.K.); aabdallah@kumc.edu (A.-O.A.)

**Keywords:** high-risk multiple myeloma, post-ASCT maintenance, doublet maintenance, lenalidomide, bortezomib

## Abstract

**Background:** Lenalidomide maintenance after autologous stem cell transplantation (ASCT) improves progression-free survival (PFS) and overall survival (OS) in multiple myeloma; however, these benefits are attenuated in high-risk multiple myeloma (HRMM). No standard post-transplant maintenance strategy is established for HRMM, and some centers employ doublet maintenance with bortezomib plus lenalidomide (VR). We evaluated outcomes with VR versus lenalidomide alone (R) in HRMM. **Methods:** We conducted a multicenter retrospective study through the US Myeloma Innovations Research Collaborative (USMIRC), including adults with HRMM who received R or VR maintenance following ASCT between January 2009 and January 2024. HRMM was defined by del(17p), t(4;14), t(14;16), or t(14;20), with or without 1q gain. PFS and OS were estimated using Kaplan–Meier methods. Median follow-up was 91 months. Baseline characteristics, induction regimens, and post-transplant response depth were well balanced between the groups. Median PFS was 51 months (95% CI, 20–NR) with VR and 36 months (95% CI, 31–56) with R (*p* > 0.05). Median OS was 103 months (95% CI, 90–NR) and 110 months (95% CI, 94–NR), respectively (*p* > 0.05). VR was associated with numerically longer PFS, although the difference was not statistically significant. No treatment-related mortality occurred within 100 days post-ASCT. **Conclusions:** In this multicenter real-world analysis of HRMM, VR maintenance did not result in statistically significant improvements in PFS or OS compared with lenalidomide alone. These findings underscore the need for prospective, risk-adapted trials incorporating novel maintenance strategies, including CD38- and BCMA-directed therapies, in high-risk disease.

## Simple Summary

Maintenance therapy plays a critical role in prolonging disease control and improving long-term outcomes in patients with high-risk multiple myeloma (HRMM). While lenalidomide maintenance following autologous stem cell transplantation (ASCT) has been shown to improve progression-free survival (PFS) and overall survival (OS) in multiple myeloma, these benefits are attenuated in patients with HRMM. Given that some centers employ doublet maintenance strategies despite the absence of a clearly established standard of care for HRMM, our multicenter analysis addresses an important gap in the current literature by comparing the efficacy and safety of doublet versus single-agent maintenance therapy, specifically bortezomib plus lenalidomide (VR) versus lenalidomide alone (R), in the post-transplant setting.

## 1. Introduction

Multiple myeloma (MM) is a clonal plasma-cell neoplasm defined by ≥10% clonal bone-marrow plasma cells or a biopsy-proven plasmacytoma plus at least one myeloma-defining event [1]. Multiple myeloma (MM) accounts for approximately 10% of hematologic malignancies [1] and remains incurable for most patients due to relapse or refractoriness to treatment [2,3]. Nevertheless, substantial survival gains have been achieved with triplet compared with quadruplet [4] induction, autologous stem cell transplant (ASCT) [1,5], and maintenance therapy [6,7]. Because relapse after ASCT is common, maintenance therapy has become a core component of long-term management, prolonging progression-free survival (PFS) [6,8] and, in some studies, overall survival (OS) [7,9]. Lenalidomide is the standard maintenance agent, supported by multiple randomized trials and a meta-analysis that showed a significant PFS benefit versus observation, with OS advantages emerging with longer follow-up [6,7,8,9,10].

Biologic heterogeneity drives outcomes in MM, and specific cytogenetic abnormalities define high-risk disease, resulting in inferior survival [11]. The International Myeloma Working Group (IMWG) and contemporary practice updates classify del(17p), t(4;14), t(14;16), t(14;20), and gain or amplification of 1q as high-risk; the presence of two or more such abnormalities is described as “double-hit” or “triple-hit” myeloma with further adverse prognosis [1,11]. Extra copies of 1q are common and show a dose-dependent adverse effect on outcomes, with amplification (≥4 copies) performing worse than simple gain [12]. A review of maintenance randomized trials revealed that lenalidomide use as maintenance led to improved PFS after ASCT; however, high-risk and ultra-high-risk cohorts continue to experience inferior outcomes compared to standard-risk patients, suggesting attenuated benefit compared with standard-risk disease [8]. Accordingly, contemporary guidance advocates risk-adapted maintenance, commonly intensifying therapy for high-risk patients by combining proteasome inhibitors and immunomodulatory agents (e.g., bortezomib plus lenalidomide) [1,2,13].

The biological rationale for adding a proteasome inhibitor, such as bortezomib or ixazomib, is compelling in high-risk MM, particularly for cytogenetic abnormalities such as del(17p) and t(4;14), which are associated with proteasome dependence. In the HOVON-65/GMMG-HD4 randomized trial, a regimen comprising bortezomib-based induction followed by bortezomib maintenance led to improved outcomes (PFS and OS) in predefined high-risk groups, including patients with del(17p) [4]. In the TOURMALINE-MM3 trial, ixazomib maintenance after ASCT prolonged PFS relative to placebo with a manageable safety profile [14]. These findings support proteasome inhibitor maintenance as a practical option in select patients with adverse cytogenetic profiles, while the detailed effects in genomically defined high-risk cohorts have yet to be clarified.

Novel antibody-based combinations have expanded the maintenance landscape in MM. In transplant-eligible patients with newly diagnosed MM, daratumumab added to VRd induction/consolidation followed by daratumumab–lenalidomide maintenance significantly reduced the risk of progression or death in the Phase 3 PERSEUS trial [15]. The Phase 2 GRIFFIN study similarly showed a more durable response and better PFS with D-VRd, with maintenance incorporating daratumumab plus lenalidomide [16,17,18]. Emerging randomized data focused specifically on the maintenance phase (AURIGA) indicate that daratumumab–lenalidomide maintenance improves measurable residual disease (MRD) negativity conversion and PFS versus lenalidomide alone in patients who remain MRD-positive post-ASCT [19]. Together, these studies support antibody-augmented maintenance as a promising direction, especially for biologically high-risk disease.

Despite these advances, there is no universally accepted standard for maintenance therapy in high-risk MM [20,21]. Clinical practice varies, and many centers intensify maintenance therapy for high-risk patients by combining lenalidomide with a proteasome inhibitor, such as bortezomib, extrapolating from HOVON-65 and risk-adapted expert guidance [1,22]. However, comparative effectiveness data specifically contrasting doublet bortezomib–lenalidomide (VR) versus lenalidomide alone (R) in high-risk patients are limited, heterogeneous, and often single-center [21,23,24]. Recent retrospective case series have reported mixed results, complicated by selection biases and variability in the definitions of high-risk cytogenetics and 1q amplification [21,24]. Multicenter, real-world analyses focused on high-risk cohorts remain scarce.

To address this gap, we conducted a multicenter retrospective study within the U.S. Myeloma Innovations Research Collaborative (USMIRC) comparing VR versus R maintenance after ASCT in high-risk MM, with outcomes including PFS and OS, and a prespecified evaluation across key cytogenetic subgroups.

## 2. Methods

We conducted a multicenter retrospective study in collaboration with the U.S. Myeloma Innovations Research Collaborative (USMIRC) to evaluate the real-world effectiveness of bortezomib plus lenalidomide (VR) versus lenalidomide monotherapy (R) as post–autologous stem cell transplant (ASCT) maintenance therapy in patients with high-risk multiple myeloma (HRMM). The study period spanned from January 2009 to January 2024 and included adult patients (≥18 years) treated across participating USMIRC centers.

High-risk multiple myeloma (HRMM) was defined based on cytogenetic abnormalities by the presence of one or more of the following abnormalities identified by fluorescence in situ hybridization (FISH): deletion 17p, t(4;14), t(14;16), or t(14;20), with or without chromosome 1q gain or amplification. This definition reflects the IMWG 2016 consensus criteria, which were contemporary to the study period (2009–2024), rather than the revised 2025 IMWG classification requiring co-occurrence of primary cytogenetic abnormalities with 1q gain/amplification or del(1p32) for high-risk designation. In addition, the threshold for del(17p) positivity varied by institution based on each center’s validated FISH laboratory cutoff, consistent with real-world practice across a 15-year study period.

Eligible patients had undergone induction and ASCT, followed by maintenance therapy with either R alone or VR doublet within 180 days of ASCT. Patients who received tandem or allogeneic transplantation or alternative maintenance regimens were excluded from the study. Patients who achieved a suboptimal response (less than a partial response) after initial induction were eligible to receive a second induction regimen before ASCT, at the discretion of the treating physician. Response assessments were performed after each induction course according to IMWG uniform response criteria.

Baseline demographic and clinical characteristics, including age, sex, race, Revised International Staging System (R-ISS) stage, cytogenetic profile, myeloma subtype, and performance status, were extracted from institutional databases. Treatment-related data included the type of induction regimen and conditioning protocol. Response assessments were performed according to the IMWG uniform response criteria. The overall response rate (ORR) was defined as the proportion of patients achieving ≥partial response (PR), and deep response was defined as ≥very good partial response (VGPR). Continuous variables were summarized using median and interquartile range (IQR), and categorical variables were summarized using counts and percentages. Comparisons were made between the VR and R groups. For continuous variables, the Wilcoxon rank-sum test was performed. Associations between categorical variables and maintenance therapy were assessed using the Pearson chi-squared test; when expected cell counts were small, Fisher’s exact test was used. The test was performed only on available data, and no data imputation was applied. The primary endpoints were progression-free survival (PFS) and overall survival (OS), measured from the date of ASCT to disease progression, death, or the last follow-up. The secondary endpoints included ORR post-induction, post-ASCT, and at 6 and 12 months following the initiation of maintenance therapy. ORR post-induction and post-ASCT were also reported as descriptive data to characterize baseline response status at the initiation of maintenance therapy. The differences in overall survival were compared based on the restricted mean survival time using the survRM2 package (version 1.0-4) in R (version 4.3.1).

Given the retrospective nature of the analysis, the requirement for informed consent was waived. All patient data were de-identified to ensure confidentiality and compliance with the Health Insurance Portability and Accountability Act (HIPAA).

## 3. Results

### 3.1. Baseline Characteristics

A total of 97 patients with high-risk cytogenetic multiple myeloma were included in the analysis. Among them, 68 (70%) received lenalidomide (R) maintenance, and 29 (30%) received bortezomib–lenalidomide (VR) doublet maintenance therapy post-transplantation.

The median age of the overall cohort was 60 years (IQR 52–67), with similar medians observed in the R (60 years, IQR 51–67) and VR (57 years, IQR 52–66) groups. Slightly more than half of the overall population was male (52%), with a higher proportion of men in the VR arm than in the R arm (62% vs. 47%) as shown in Table 1.

The majority of patients were White (69%), with equal representation across the R (69%) and VR (69%) groups. African American patients represented 23% of the total cohort (24% R and 21% VR). Smaller proportions of patients were identified as Asian (3.1% overall; higher in VR at 6.9% vs. 1.5% in R), Hispanic (2.1%, all in R), or Other (3.1%).

Most patients had good functional status at baseline. ECOG performance status (PS) 0 or 1 was observed in 89% of patients (87% R, 97% VR). A small proportion had PS 2 (9.3% overall; more common in the R group [12%] than in the VR group [3.4%]). Only one patient had a PS of 3 (R group).

Based on the Revised International Staging System (R-ISS), stage II disease predominated (61% overall; 54% R and 76% VR). Stage III disease was observed in 29% of patients overall, with a higher proportion in the R group (32% vs. 21% in the VR group). Stage I disease was less common (7.2% overall), and three patients had missing staging data.

The distribution of myeloma subtypes was comparable between the two maintenance groups. IgG kappa was the most common subtype overall, accounting for 38.1% of patients (33.8% in the R group vs. 48.2% in the VR group). This was followed by IgG lambda in 22.6% (25% vs. 17.2%) and IgA kappa in 20.6% (19.1% vs. 24.1%) of patients in the R and VR groups, respectively. IgA lambda was identified in 5.1% of patients (5.9% vs. 3.4%), whereas free light chain (FLC) disease accounted for a smaller proportion: free kappa in 7.2% (8.8% vs. 3.4%) and free lambda in 5.1% (7.3% vs. 0%) of patients. Only one patient (1.0%) presented with non-secretory myeloma, occurring exclusively in the VR cohort. Overall, the distribution of immunoglobulin isotypes did not significantly differ between the two maintenance groups (*p* > 0.05).

Among the 97 patients with high-risk multiple myeloma, the distribution of cytogenetic high-risk subgroups was comparable between the two arms (*p* = 0.60). Double- or triple-hit abnormalities were observed in 12% of the overall patient population, with similar frequencies in the R (12%) and VR (14%) groups. Translocations involving t(14,16)/t(14,20) occurred in 9.3% of patients and were more frequent in the R cohort (12%) than the VR cohort (3.4%). Notably, 30% of the overall patient population had t(4,14), and this was similarly distributed between the groups. TP53 abnormalities or del (17p) were the most common feature, presented in 48% of patients overall, with no meaningful difference between the cohorts.

### 3.2. Response to Induction Therapy

Overall response rates were assessed in all subjects, including those who received one induction therapy and those who received a second induction. In this cohort with a single induction, 91% of patients achieved an objective response, including 88% in the R group and 97% in the VR group. The depth of response varied modestly across groups. Among patients in the R cohort, 15% achieved a complete response (CR), 2.9% achieved a stringent complete response (sCR), 24% achieved a partial response (PR), and 47% achieved a very good partial response (VGPR). In comparison, patients in the VR group demonstrated numerically higher rates of deep responses: 28% achieved CR, 10% achieved sCR, 28% achieved PR, and 31% achieved VGPR. Progressive disease (PD) was observed in two patients (2.9%) in the R group and none in the VR group, see Table 2 for reference.

A subset of patients received second induction therapy due to suboptimal response (less than partial response) after initial induction. Among these patients, response rates were lower overall due to a large proportion of patients classified as not evaluable. In this subset, 12% of patients in the R group and 6.9% in the VR group achieved a measurable response. Within the R cohort, two patients (2.9%) achieved CR, one (1.5%) achieved sCR, one (1.5%) achieved PR, and seven (10%) achieved VGPR. In the VR cohort, one patient (3.4%) achieved PR, and one (3.4%) achieved VGPR, with no CR or sCR reported.

### 3.3. Post-ASCT Response

After ASCT but prior to maintenance, the ORR remained high (94% overall; 93% R, 97% VR) as shown in Table 3. In the R arm, 38%, 13%, and 38% of patients achieved CR, sCR, and VGPR, respectively. In the VR arm, 34%, 17%, and 34% of patients achieved CR, sCR, and VGPR, respectively. PRs were observed in 4.4% of R and 10% of VR patients. Non-evaluable responses were rare (<6%).

### 3.4. Response at 6 Months Post-Maintenance

At 6 months post-maintenance initiation, the responses were durable across both groups. In the R cohort, CR was achieved in 25 patients (37%), sCR in 11 (16%), VGPR in 15 (22%), and PR in none of the patients. In the VR cohort, CR was observed in eight patients (28%), sCR in four (14%), VGPR in six (21%), and PR in one (3%). Progressive disease occurred in nine (13%) and five (17%) patients on R and VR, respectively. Treatment discontinuation due to adverse events was observed in only one patient, Table 4 demonstrates the same.

### 3.5. Response at 12 Months Post-Maintenance

By 12 months, the depth of response remained broadly similar between the groups. In the R arm, 24 patients (35%) achieved CR, 9 (13%) sCR, 10 (15%) VGPR, and none achieved PR. In the VR group, CR was observed in six patients (21%), sCR in three (10%), VGPR in four (14%), and PR in one (3%). PD occurred in 10 (15%) and 5 (17%) patients in the R and VR arms, respectively. Treatment discontinuation due to toxicity occurred in one patient who received VR. A higher proportion of patients with VR were unevaluable at 12 months (17% vs. 8.8%).

### 3.6. Survival Outcomes

**Figure 1:**
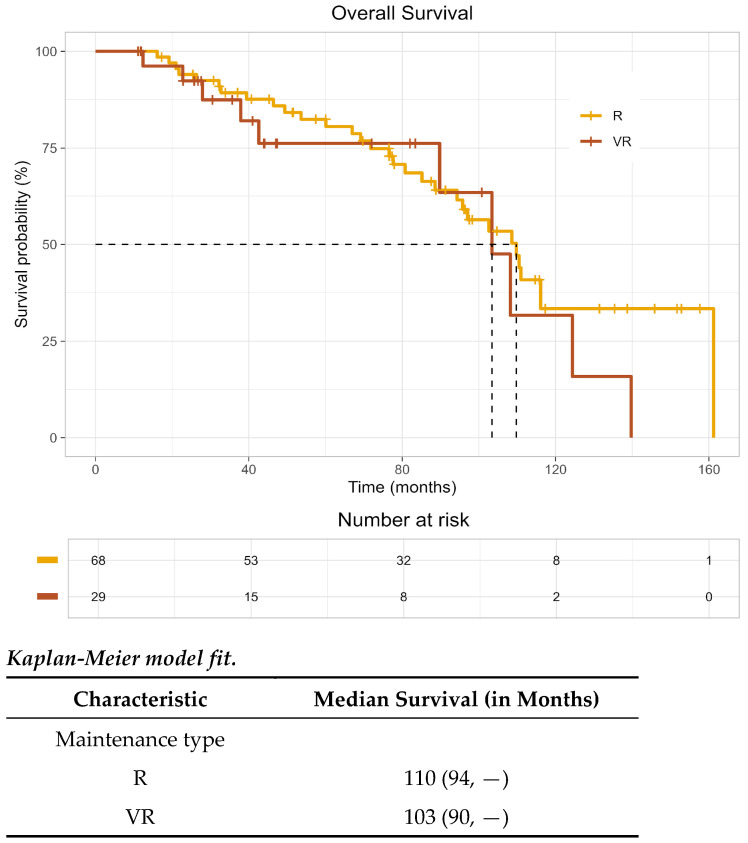
Overall survival post-maintenance with VR (bortezomib–lenalidomide) versus R (lenalidomide).

**Figure 2:**
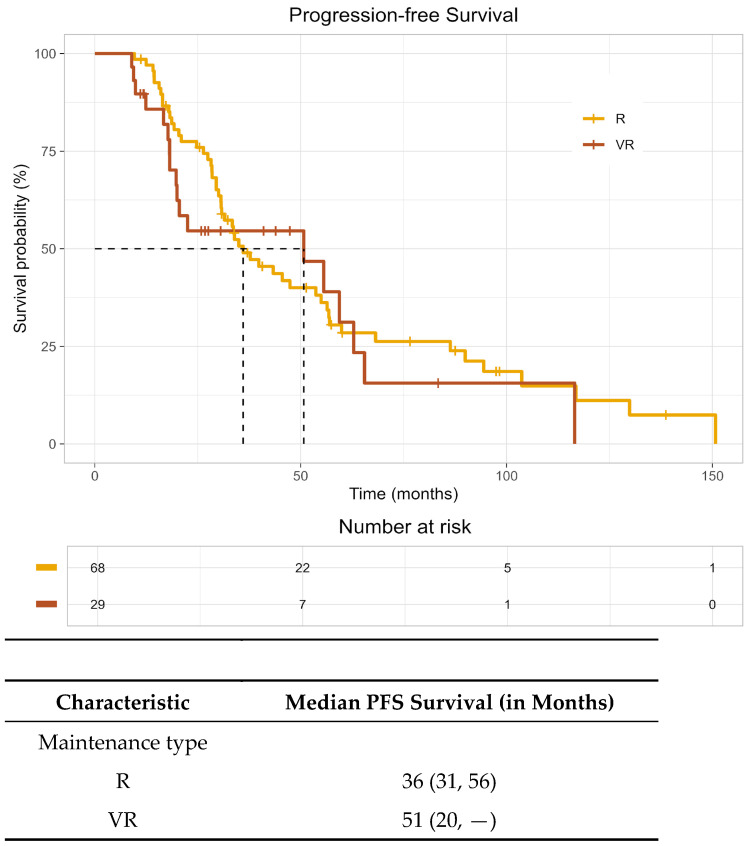
PFS post-maintenance with VR (bortezomib–lenalidomide) versus R (lenalidomide).

On Kaplan–Meier analysis, median overall survival (OS) was estimated at 110 months (95% CI: 94, not reached) in the R group and 103 months (95% CI: 90, not reached) in the VR group, demonstrated in Figure 1. As the KM curve crosses and the proportional hazard assumption is violated, instead of calculating the hazard ratio, we computed the restricted mean survival time. For comparison, at the follow-up of 140 months, the difference in restricted mean survival time was 5.3 months (95% CI: −16.4, 27.0), and the *p*-value was 0.634. The median progression-free survival (PFS) in the R group was 36 months (95% CI: 31–56); the median PFS in the VR group was 51 months (95% CI: 20, not reached), as shown in Figure 2. At the follow-up of 117 months, the difference in restricted mean PFS time was 3.9 months (95% CI: −14.1, 21.9), and the *p*-value was 0.672. The wide confidence interval was due to the small sample size. As such, no conclusions regarding a PFS difference between groups can be made, and continued follow-up is warranted.

## 4. Discussion

In this multicenter real-world HRMM cohort, median PFS in the lenalidomide-alone (R) group was 36 months (95% CI: 31–56). Median PFS in the bortezomib–lenalidomide (VR) group was 51 months (95% CI: 20–NR). The wide confidence interval for PFS in the VR arm reflects the limited sample size and warrants cautious interpretation. Restricted mean survival time (RMST) analysis, which estimates the average event-free survival up to a specified time horizon, showed a PFS difference of 3.9 months (95% CI: −14.1, 21.9; *p* = 0.672). Median OS was estimated at 110 months (95% CI: 94, not reached) in the R group and 103 months (95% CI: 90, not reached) in the VR group. Responses at 6 and 12 months were durable in both arms, and early treatment-related mortality was not observed in either group.

This finding is consistent with the benefit observed in trials demonstrating that post-transplant exposure to proteasome inhibitors improves disease control and survival outcomes. In HOVON-65/GMMG-HD4, a bortezomib-containing regimen that included bortezomib maintenance improved PFS and showed OS advantages in the overall population, as well as in predefined adverse-risk subsets, such as del(17p), when compared to a thalidomide-based regimen [4,22]. Overall, while these historical results support adding a proteasome inhibitor during the maintenance period in adverse biology, it is important to acknowledge that the HOVON-65/GMMG-HD4 trial utilized older comparators and preceded current salvage options [4,22,25].

Data from the randomized, open-label Phase 2 FORTE trial [26] show that adding carfilzomib to lenalidomide (KR) during maintenance improves progression-free survival after ASCT compared with lenalidomide (R) alone. Utilizing randomized maintenance intensification (defined as adding carfilzomib to lenalidomide), KR maintenance reduced the risk of progression or death versus R (HR 0.64, 95% CI 0.44–0.94; 3-year PFS 75% vs. 65%) [26], providing supporting evidence that doublet maintenance can improve survival indices after ASCT. Beyond the overall maintenance effect, it is important to determine whether this benefit persists across cytogenetic risk strata. In a prespecified cytogenetic analysis of the FORTE trial [27], a downtrend in PFS was noted with an increasing high-risk cytogenetic burden, revealing 80%, 68%, and 53% 3-year PFS with zero, one, and two or more high-risk cytogenetic abnormalities (HRCA), respectively, indicating an increasing risk of multi-hit disease. Exploratory subgroup readouts suggested that the KR maintenance advantage extended to canonical lesions such as del(17p) and t(4;14), whereas 1q amplification remained particularly adverse despite intensified therapy. These observations align with our cohort, in which intensified maintenance therapy favored PFS without an early OS difference, and further show the need for more research in patients with MM with ultra-high-risk biology. Our findings are also consistent with the Nordic Myeloma Study Group Phase 2 trial, which employed cytogenetic risk-based stratification, assigning high-risk patients to ixazomib–lenalidomide maintenance and standard/low-risk patients to lenalidomide alone. Notably, high-risk patients receiving doublet maintenance achieved similar PFS to standard/low-risk patients on lenalidomide alone (66% progression-free at 2 years; *p* = 0.395), supporting the rationale that intensified maintenance may mitigate the adverse prognostic impact of high-risk cytogenetics [28].

Nonetheless, antibody-based combinations are reshaping the expectations of transplant-eligible patients with multiple myeloma. The PERSEUS trial demonstrated that adding daratumumab to VRd induction and consolidation, followed by daratumumab–lenalidomide maintenance, significantly reduced the risk of progression or death compared to VRd followed by lenalidomide alone in transplant-eligible patients [15]. In the randomized controlled GRIFFIN Phase 2 Trial, which explored the addition of daratumumab to lenalidomide, bortezomib, and dexamethasone (D-RVd) versus lenalidomide, bortezomib, and dexamethasone only, the D-RVd study arm performed better in terms of PFS than the RVd arm [16,18]. Focusing specifically on the maintenance phase, the AURIGA trial found that daratumumab–lenalidomide improved MRD negativity conversion and PFS compared with lenalidomide alone in patients who remained MRD-positive post-ASCT [19]. When evaluated side-by-side, these data support antibody-augmented maintenance therapy as a promising direction, particularly for biologically adverse diseases. Beyond CD38-targeted antibodies, BCMA-targeted bispecific T-cell engagers are being evaluated as maintenance therapy in the frontline setting, including the ongoing Phase 3 MajesTEC-4 (EMN30) trial evaluating teclistamab versus lenalidomide maintenance after autologous transplant in newly diagnosed patients.

The absence of an early OS difference is consistent with the contemporary relapse rates. High-activity salvage regimens at first relapse, including CD38–antibody triplets, can attenuate early OS separation even when frontline or maintenance strategies prolong PFS, and randomized BCMA-directed cell-therapy trials have further improved PFS after first progression. Updated final analyses from POLLUX [29] and CASTOR [30] reported OS improvements with daratumumab-containing salvage triplets, and KarMMa-331 and CARTITUDE-432 demonstrated superior PFS for ide-cel and cilta-cel versus standard regimens in patients who had received prior therapy, which may delay OS divergence between upfront maintenance strategies [31,32].

Real-world comparisons illustrate how outcomes reflect both disease biology and selection, with two large retrospective comparisons reaching different conclusions when focusing on high-risk patients after ASCT. In a real-world, multicenter CIBMTR analysis restricted to high-risk cytogenetics (*n* = 503) [24], lenalidomide maintenance was associated with better 2-year PFS than bortezomib-based maintenance, although the bortezomib cohort had more adverse baseline features and included both monotherapy and combination regimens [24]. In contrast, a trial-derived retrospective comparison pooling two GMMG Phase 3 studies, not limited to high-risk patients, found no significant difference in PFS or OS between lenalidomide and bortezomib maintenance therapy after ASCT [23].

In this multicenter retrospective analysis, VR maintenance was associated with a numerically longer median PFS compared with R alone, though this difference did not reach statistical significance. These hypothesis-generating findings support further investigation of doublet maintenance strategies in high-risk MM through prospective, adequately powered trials. Given the evolving treatment landscape, future studies should incorporate MRD-guided approaches and novel agents, including CD38- and BCMA-directed therapies.

### Strengths, Limitations, and Future Directions

This retrospective analysis is subject to selection bias and unmeasured confounding. The small sample size (*n* = 97), particularly in the VR cohort (n = 29), limits the statistical power to detect modest differences. Granular cytogenetic stratification, including differentiation of 1q gain versus amplification and institutional FISH-specific thresholds, was not feasible despite potential prognostic relevance. MRD data were unavailable, limiting biological interpretation and comparison with contemporary MRD-guided maintenance studies. Maintenance dosing, duration, and toxicity assessment were not standardized across centers, and granular dosing data were not systematically captured in this retrospective analysis, introducing treatment heterogeneity that may influence PFS. Granular toxicity data, including graded neuropathy assessments, were not systematically captured. No treatment-related mortality occurred within 100 days post-ASCT, and treatment discontinuation due to adverse events was uncommon. Additionally, a subset of patients required a second induction due to suboptimal initial response, which may reflect more refractory disease biology; however, the small numbers preclude meaningful subgroup analysis. In addition, the prolonged study period encompasses substantial advances in salvage therapy, including CD38-directed antibodies and BCMA-targeted agents, which may attenuate OS differences between maintenance strategies. Our high-risk definition was based on the 2016 IMWG consensus criteria, which classified del(17p), t(4;14), t(14;16), and t(14;20) as high-risk abnormalities regardless of co-occurring lesions. The recently updated 2025 IMWG criteria now require co-existence of these abnormalities with 1q gain/amplification or del(1p32) for high-risk classification. While our definition was appropriate for the study period, future analyses should consider the revised risk stratification framework.

Key strengths include the multicenter USMIRC collaboration, which enhances generalizability, and the restriction of the cohort to patients with predefined high-risk cytogenetic abnormalities, defined by IMWG criteria. Long-term follow-up supports the robustness of PFS and OS estimates, and uniform application of IMWG response criteria improves consistency of response assessment. Baseline characteristics were well balanced between the groups. Importantly, this study provides real-world data addressing an area with limited prospective evidence in high-risk multiple myeloma.

For future directions, it would be beneficial for prospective trials to test maintenance intensification strategies that incorporate anti-CD38 antibodies and emerging BCMA-directed therapies, with predefined analyses targeting high-risk cytogenetics. Given the scale of prolonged maintenance, future studies should address cost-effectiveness, patient-reported outcomes, systematic adverse event monitoring, and long-term toxicity.

## 5. Conclusions

In this multicenter retrospective analysis conducted through the U.S. Myeloma Innovations Research Collaborative (USMIRC), doublet maintenance with bortezomib plus lenalidomide (VR) was associated with a numerically longer median PFS (51 vs. 36 months) compared with lenalidomide alone (R) in patients with high-risk multiple myeloma following ASCT; however, this difference did not reach statistical significance. Median OS was comparable between the two groups (103 vs. 110 months), and no treatment-related mortality was observed within 100 days post-ASCT. Response rates at 6- and 12-month post-maintenance were durable across both arms.

These findings highlight the persistent unmet need for effective maintenance strategies in high-risk multiple myeloma. While the addition of bortezomib to lenalidomide maintenance did not demonstrate a statistically significant survival benefit in our cohort, the limited sample size and wide confidence intervals warrant cautious interpretation and emphasize the need for adequately powered prospective trials. The evolving treatment landscape, including CD38-directed antibodies and BCMA-targeted therapies, offers promising avenues for maintenance intensification in this population. Future studies should incorporate MRD-guided approaches, risk-adapted stratification aligned with updated IMWG criteria, and systematic assessment of long-term safety and patient-reported outcomes to optimize post-transplant maintenance in high-risk disease.

## Figures and Tables

**Table 1:** Baseline demographics and disease characteristics.

Characteristic	Overall N = 97 ^1^	R N = 68 ^1^	VR N = 29 ^1^	*p*-Value ^2^
**Age**	60 (52, 67)	60 (51, 67)	57 (52, 66)	0.8
Unknown	3	1	2	
**Gender**				0.2
F	47 (48%)	36 (53%)	11 (38%)	
M	50 (52%)	32 (47%)	18 (62%)	
**Race**				0.6
AA	22 (23%)	16 (24%)	6 (21%)	
Asian	3 (3.1%)	1 (1.5%)	2 (6.9%)	
Hispanic	2 (2.1%)	2 (2.9%)	0 (0%)	
Other	3 (3.1%)	2 (2.9%)	1 (3.4%)	
W	67 (69%)	47 (69%)	20 (69%)	
**PS**				0.5
0	15 (15%)	9 (13%)	6 (21%)	
1	72 (74%)	50 (74%)	22 (76%)	
2	9 (9.3%)	8 (12%)	1 (3.4%)	
3	1 (1.0%)	1 (1.5%)	0 (0%)	
**Stage R-ISS**				0.3
1	7 (7.2%)	6 (8.8%)	1 (3.4%)	
2	59 (61%)	37 (54%)	22 (76%)	
3	28 (29%)	22 (32%)	6 (21%)	
Missing	3 (3.1%)	3 (4.4%)	0 (0%)	
**Type of Myeloma**				0.3
Free Kappa	7 (7.2%)	6 (8.8%)	1 (3.4%)	
Free lambda	5 (5.1%)	5 (7.3%)	0 (0%)	
IgA Kappa	20 (20.6%)	13 (19.1%)	7 (24.1%)	
IgA Lambda	5 (5.1%)	4 (5.9%)	1 (3.4%)	
IgG Kappa	37 (38.1%)	23 (33.8%)	14 (48.2%)	
IgG Lambda	22 (22.6%)	17 (25%)	5 (17.2%)	
non-secretory	1 (1.0%)	0 (0%)	1 (3.4%)	
**Cytogenetics Subgroup**				0.6
Double/Triple Hit	12 (12%)	8 (12%)	4 (14%)	
t(14;16)/t(14;20)	9 (9.3%)	8 (12%)	1 (3.4%)	
t(4;14)	29 (30%)	19 (28%)	10 (34%)	
TP53/del(17p)	47 (48%)	33 (49%)	14 (48%)	

^1^ Median (Q1, Q3); n (%). ^2^ Wilcoxon rank-sum test; Pearson’s chi-squared test; Fisher’s exact test.

**Table 2:** Response to induction therapy.

Characteristic	Overall N = 97 ^1^	R N = 68 ^1^	VR N = 29 ^1^	*p*-Value ^2^
**ORR post 1st Induction**				0.3
N	9 (9.3%)	8 (12%)	1 (3%)	
Y	88 (91%)	60 (88%)	28 (97%)	
**ORR (Type of Response)**				0.2
CR	18 (19%)	10 (15%)	8 (28%)	
PD	2 (2.1%)	2 (2.9%)	0 (0%)	
PR	24 (25%)	16 (24%)	8 (28%)	
sCR	5 (5.2%)	2 (2.9%)	3 (10%)	
SD	1 (1.0%)	1 (1.5%)	0 (0%)	
VGPR	41 (42%)	32 (47%)	9 (31%)	
N/A	6 (6.2%)	5 (7.4%)	1 (3.4%)	
**ORR post 2nd Induction**				0.2
Y	12 (12%)	10 (15%)	2 (6.9%)	
N	9 (9.3%)	4 (5.9%)	5 (17%)	
r	1 (1.0%)	1 (1.5%)	0 (0%)	
N/A	75 (77%)	53 (78%)	22 (76%)	
**ORR (Type of Response)**				0.6
CR	2 (2.1%)	2 (2.9%)	0 (0%)	
PR	2 (2.1%)	1 (1.5%)	1 (3.4%)	
sCR	1 (1.0%)	1 (1.5%)	0 (0%)	
VGPR	8 (8.2%)	7 (10%)	1 (3.4%)	
N/A	84 (87%)	57 (84%)	27 (93%)	

^1^ n (%). ^2^ Fisher’s exact test; Abbreviations: CR, complete response; sCR, stringent complete response; VGPR, very good partial response; PR, partial response; SD, stable disease; PD, progressive disease; NE, not evaluable; Y, yes; N, no; N/A, not applicable; r, response maintained.

**Table 3:** Response post-ASCT.

Characteristic	Overall N = 97 ^1^	R N = 68 ^1^	VR N = 29 ^1^	*p*-Value ^2^
**ASCT ORR**	91 (94%)	63 (93%)	28 (97%)	0.7
**Post ASCT ORR (Type of Response)**				0.7
CR	36 (37%)	26 (38%)	10 (34%)	
PR	6 (6.2%)	3 (4.4%)	3 (10%)	
sCR	14 (14%)	9 (13%)	5 (17%)	
VGPR	36 (37%)	26 (38%)	10 (34%)	
N/A	5 (5.2%)	4 (5.9%)	1 (3.4%)	

^1^ n (%); ^2^ Fisher’s exact test; Abbreviations: CR, complete response; PR, partial response; sCR, stringent complete response; VGPR, very good partial response; N/A, not applicable.

**Table 4:** Response at 6 months and 12 months post-maintenance.

Characteristic	Overall N = 97 ^1^	R N = 68 ^1^	VR N = 29 ^1^
**ORR post-maintenance 6 months**			
CR	33 (34%)	25 (37%)	8 (28%)
PD	14 (14%)	9 (13%)	5 (17%)
PR	1 (1.0%)	0 (0%)	1 (3.4%)
sCR	15 (15%)	11 (16%)	4 (14%)
SD	2 (2.1%)	2 (2.9%)	0 (0%)
VGPR	21 (22%)	15 (22%)	6 (21%)
Y	10 (10%)	6 (8.8%)	4 (14%)
Y, stopped due to AE	1 (1.0%)	0 (0%)	1 (3.4%)
**ORR post-maintenance 12 months**			
CR	30 (31%)	24 (35%)	6 (21%)
N	2 (2.1%)	2 (2.9%)	0 (0%)
N/A	11 (11%)	6 (8.8%)	5 (17%)
PD	15 (15%)	10 (15%)	5 (17%)
PR	1 (1.0%)	0 (0%)	1 (3.4%)
sCR	12 (12%)	9 (13%)	3 (10%)
SD	2 (2.1%)	2 (2.9%)	0 (0%)
VGPR	14 (14%)	10 (15%)	4 (14%)
Y	8 (8.2%)	4 (5.9%)	4 (14%)
Stopped due to AE	1 (1.0%)	0 (0%)	1 (3.4%)
unknown	1 (1.0%)	1 (1.5%)	0 (0%)

^1^ n (%). Abbreviations: CR, complete response; sCR, stringent complete response; VGPR, very good partial response; PR, partial response; SD, stable disease; PD, progressive disease; NE, not evaluable; Y, yes; N, no; N/A, not applicable.

## Data Availability

The data presented in this study are available upon request from the corresponding author.

## References

[1] Rajkumar S.V. (2024). Multiple myeloma: 2024 update on diagnosis, risk-stratification, and management. Am. J. Hematol..

[2] Malard F., Neri P., Bahlis N.J., Terpos E., Moukalled N., Hungria V.T.M., Manier S., Mohty M. (2024). Multiple myeloma. Nat. Rev. Dis. Prim..

[3] Durie B.G.M., Hoering A., Abidi M.H., Rajkumar S.V., Epstein J., Kahanic S.P., Thakuri M., Reu F., Reynolds C.M., Sexton R. (2017). Bortezomib with lenalidomide and dexamethasone versus lenalidomide and dexamethasone alone in patients with newly diagnosed myeloma without intent for immediate autologous stem-cell transplant (SWOG S0777): A randomised, open-label, phase 3 trial. Lancet.

[4] Sonneveld P., Schmidt-Wolf I.G., van der Holt B., el Jarari L., Bertsch U., Salwender H., Zweegman S., Vellenga E., Broyl A., Blau I.W. (2012). Bortezomib Induction and Maintenance Treatment in Patients With Newly Diagnosed Multiple Myeloma: Results of the Randomized Phase III HOVON-65/GMMG-HD4 Trial. J. Clin. Oncol..

[5] Attal M., Harousseau J.-L., Stoppa A.-M., Sotto J.-J., Fuzibet J.-G., Rossi J.-F., Casassus P., Maisonneuve H., Facon T., Ifrah N. (1996). A Prospective, Randomized Trial of Autologous Bone Marrow Transplantation and Chemotherapy in Multiple Myeloma. N. Engl. J. Med..

[6] Attal M., Lauwers-Cances V., Marit G., Caillot D., Moreau P., Facon T., Stoppa A.M., Hulin C., Benboubker L., Garderet L. (2012). Lenalidomide maintenance after stem-cell transplantation for multiple myeloma. N. Engl. J. Med..

[7] McCarthy P.L., Owzar K., Hofmeister C.C., Hurd D.D., Hassoun H., Richardson P.G., Giralt S., Stadtmauer E.A., Weisdorf D.J., Vij R. (2012). Lenalidomide after stem-cell transplantation for multiple myeloma. N. Engl. J. Med..

[8] Jackson G.H., Davies F.E., Pawlyn C., Cairns D.A., Striha A., Collett C., Hockaday A., Jones J.R., Kishore B., Garg M. (2019). Lenalidomide maintenance versus observation for patients with newly diagnosed multiple myeloma (Myeloma XI): A multicentre, open-label, randomised, phase 3 trial. Lancet Oncol..

[9] McCarthy P.L., Holstein S.A., Petrucci M.T., Richardson P.G., Hulin C., Tosi P., Bringhen S., Musto P., Anderson K.C., Caillot D. (2017). Lenalidomide maintenance after autologous stem-cell transplantation in newly diagnosed multiple myeloma: A meta-analysis. J. Clin. Oncol..

[10] Palumbo A., Cavallo F., Gay F., Di Raimondo F., Ben Yehuda D., Petrucci M.T., Pezzatti S., Caravita T., Cerber V., Liberati A.M. (2014). Autologous transplantation and maintenance therapy in multiple myeloma. N. Engl. J. Med..

[11] Sonneveld P., Avet-Loiseau H., Lonial S., Usmani S., Siegel D., Anderson K.C., Chng W.-J., Moreau P., Attal M., Kyle R.A. (2016). Treatment of multiple myeloma with high-risk cytogenetics: A consensus of the International Myeloma Working Group. Blood.

[12] Weinhold N., Salwender H.J., Cairns D.A., Raab M.S., Waldron G., Blau I.W., Bertsch U., Hielscher T., Morgan G.J., Jauch A. (2021). Chromosome 1q21 abnormalities refine outcome prediction in patients with multiple myeloma—A meta-analysis of 2,596 trial patients. Haematologica.

[13] Zhang S., Kulkarni A.A., Xu B., Chu H., Kourelis T., Go R.S., Wang M.L., Bachanova V., Wang Y. (2020). Bortezomib-based consolidation or maintenance therapy for multiple myeloma: A meta-analysis. Blood Cancer J..

[14] Dimopoulos M.A., Gay F., Schjesvold F., Beksac M., Hajek R., Weisel K.C., Goldschmidt H., Maisnar V., Moreau P., Min C.K. (2019). Oral ixazomib maintenance following autologous stem cell transplantation (TOURMALINE-MM3): A double-blind, randomised, placebo-controlled phase 3 trial. Lancet.

[15] Sonneveld P., Dimopoulos M.A., Boccadoro M., Quach H., Ho P.J., Beksac M., Hulin C., Antonioli E., Leleu X., Mangiacavalli S. (2024). Daratumumab, Bortezomib, Lenalidomide, and Dexamethasone for Multiple Myeloma. N. Engl. J. Med..

[16] Chari A., Kaufman J.L., Laubach J., Sborov D.W., Reeves B., Rodriguez C., Silbermann R., Costa L.J., Anderson L.D., Nathwani N. (2024). Daratumumab in transplant-eligible patients with newly diagn osed multiple myeloma: Final analysis of clinically relevant subgroups in GRIFFIN. Blood Cancer J..

[17] Voorhees P.M., Sborov D.W., Laubach J., Kaufman J.L., Reeves B., Rodriguez C., Chari A., Silbermann R., Costa L.J., Anderson L.D. (2023). Addition of daratumumab to lenalidomide, bortezomib, and dexamethasone for transplantation-eligible patients with newly diagnosed multiple myeloma (GRIFFIN): Final analysis of an open-label, randomised, phase 2 trial. Lancet Haematol..

[18] Voorhees P.M., Kaufman J.L., Laubach J.P., Sborov D.W., Reeves B., Rodriguez C., Chari A., Silbermann R., Costa L.J., Anderson L.D. (2020). Daratumumab, lenalidomide, bortezomib, and dexamethasone for transplant-eligible newly diagnosed multiple myeloma: The GRIFFIN trial. Blood.

[19] Badros A., Foster L., Anderson L.D., Chaulagain C.P., Pettijohn E., Cowan A.J., Costello C., Larson S., Sborov D.W., Shain K.H. (2025). Daratumumab with lenalidomide as maintenance after transplant in newly diagnosed multiple myeloma: The AURIGA study. Blood.

[20] Hwang A., Hayden P., Pawlyn C., McLornan D., Garderet L. (2024). The role of maintenance therapy following autologous stem cell transplantation in newly diagnosed multiple myeloma: Considerations on behalf of the Chronic Malignancies Working Party of the EBMT. Br. J. Haematol..

[21] Gu X., Tang W., Zhang L., Zheng Y., Pan L., Niu T. (2024). Maintenance therapy for cytogenetically high-risk multiple myeloma: Landscape in the era of novel drugs. Clin. Exp. Med..

[22] Scheid C., Sonneveld P., Schmidt-Wolf I.G., van der Holt B., Jarari L.E., Bertsch U., Salwender H., Zweegman S., Blau I.W., Vellenga E. (2013). Bortezomib before and after autologous stem cell transplantation overcomes the negative prognostic impact of renal impairment in newly diagnosed multiple myeloma: A subgroup analysis from the HOVON-65/GMMG-HD4 trial. Haematologica.

[23] Baertsch M.-A., Mai E.K., Hielscher T., Bertsch U., Salwender H.J., Munder M., Fuhrmann S., Dührsen U., Brossart P., Neben K. (2021). Lenalidomide versus bortezomib maintenance after frontline autologous stem cell transplantation for multiple myeloma. Blood Cancer J..

[24] Bumma N., Dhakal B., Fraser R., Estrada-Merly N., Anderson K., Freytes C.O., Hildebrandt G.C., Holmberg L., Krem M.M., Lee C. (2023). Impact of bortezomib-based versus lenalidomide maintenance therapy on outcomes of patients with high-risk multiple myeloma. Cancer.

[25] Goldschmidt H., Lokhorst H.M., Mai E.K., Van Der Holt B., Blau I.W., Zweegman S., Weisel K.C., Vellenga E., Pfreundschuh M., Kersten M.J. (2018). Bortezomib before and after high-dose therapy in myeloma: Long-term results from the phase III HOVON-65/GMMG-HD4 trial. Leukemia.

[26] Gay F., Musto P., Rota-Scalabrini D., Bertamini L., Belotti A., Galli M., Offidani M., Zamagni E., Ledda A., Grasso M. (2021). Carfilzomib with cyclophosphamide and dexamethasone or lenalidomide and dexamethasone plus autologous transplantation or carfilzomib plus lenalidomide and dexamethasone, followed by maintenance with carfilzomib plus lenalidomide or lenalidomide alone for patients with newly diagnosed multiple myeloma (FORTE): A randomised, open-label, phase 2 trial. Lancet Oncol..

[27] Mina R., Musto P., Rota-Scalabrini D., Paris L., Gamberi B., Palmas A., Aquino S., de Fabritiis P., Giuliani N., De Rosa L. (2023). Carfilzomib induction, consolidation, and maintenance with or without autologous stem-cell transplantation in patients with newly diagnosed multiple myeloma: Pre-planned cytogenetic subgroup analysis of the randomised, phase 2 FORTE trial. Lancet Oncol..

[28] Partanen A., Waage A., Peceliunas V., Schjesvold F., Anttila P., Säily M., Uttervall K., Putkonen M., Carlson K., Haukas E. (2024). Ixazomib, Lenalidomide, and Dexamethasone (IRD) Treatment With Cytogenetic Risk-Based Maintenance in Transplant-Eligible Myeloma: A Phase 2 Multicenter Study by the Nordic Myeloma Study Group. Cancers.

[29] Dimopoulos M.A., Oriol A., Nahi H., San-Miguel J., Bahlis N.J., Usmani S.Z., Rabin N., Orlowski R.Z., Suzuki K., Plesner T. (2023). Overall Survival With Daratumumab, Lenalidomide, and Dexamethasone in Previously Treated Multiple Myeloma (POLLUX): A Randomized, Open-Label, Phase III Trial. J. Clin. Oncol..

[30] Sonneveld P., Chanan-Khan A., Weisel K., Nooka A.K., Masszi T., Beksac M., Spicka I., Hungria V., Munder M., Mateos M.-V. (2023). Overall Survival With Daratumumab, Bortezomib, and Dexamethasone in Previously Treated Multiple Myeloma (CASTOR): A Randomized, Open-Label, Phase III Trial. J. Clin. Oncol..

[31] Rodriguez-Otero P., Ailawadhi S., Arnulf B., Patel K., Cavo M., Nooka A.K., Manier S., Callander N., Costa L.J., Vij R. (2023). Ide-cel or Standard Regimens in Relapsed and Refractory Multiple Myeloma. N. Engl. J. Med..

[32] San-Miguel J., Dhakal B., Yong K., Spencer A., Anguille S., Mateos M.-V., de Larrea C.F., Martínez-López J., Moreau P., Touzeau C. (2023). Cilta-cel or Standard Care in Lenalidomide-Refractory Multiple Myeloma. N. Engl. J. Med..

